# Malus’s law-enhanced Michelson interferometer with PSO-based calibration and data-driven error compensation for medical micro-displacement measurement

**DOI:** 10.3389/frai.2026.1831527

**Published:** 2026-05-21

**Authors:** Yibo Wang, Yuetong Lv, Luyang Xie, Shilian Dong

**Affiliations:** 1 School of Second Clinical Hospital, Wuhan University, Wuhan, China; 2 College of Life Science and Technology, Huazhong University of Science and Technology, Wuhan, Hubei, China; 3 School of Physical Science and Technology, Wuhan University, Wuhan, China

**Keywords:** Gaussian process regression, Malus’s law, medical-engineering integration, Michelson interferometer, micro-displacement measurement, particle swarm optimization

## Abstract

**Background:**

High-precision micro-displacement measurement is crucial for applications ranging from semiconductor manufacturing to biomedical diagnostics. However, conventional Michelson interferometry is fundamentally limited by the light source’s coherence length and the inefficiency of manual fringe counting.

**Methods:**

This study introduces a modified Michelson interferometer that operates on Malus’s law. The design converts the linear micro-displacement of a movable mirror into the rotation angle of a polarizer via mechanical coupling, which shifts the measurement principle from interference fringe observation to intensity modulation. This conversion establishes a quantitative relationship between displacement and light intensity, enabling automatic photoelectric detection. Additionally, an intelligent processing module was developed to support this hardware. This module includes a Particle Swarm Optimization (PSO) algorithm for the joint calibration of hardware parameters, an analytic inverse mapping for primary displacement computation, and a Gaussian Process Regression (GPR) model to compensate for residual instrument errors while providing per-measurement uncertainty bounds.

**Results:**

The proposed system successfully overcomes the coherence-length constraints of traditional interferometry. The integration of structural modifications, intelligent parameter calibration, and data-driven error compensation establishes a novel paradigm for measuring micro-displacement.

**Conclusion:**

The Malus’s law-enhanced Michelson interferometer provides a robust and automated alternative to conventional systems. The technology demonstrates significant potential for biomedical micro-displacement monitoring, particularly for the non-contact acquisition of physiological signals in clinical settings.

## 1 Introduction

Micro-displacement, a physical quantity characterizing positional changes at the microscopic scale, is pivotal to advancing modern industry and cutting-edge technologies through high-precision measurement. In fields such as ultra-precision machining and semiconductor manufacturing, the accuracy of micro-displacement measurement exerts a direct influence on product performance. For instance, minor positional deviations between photomasks and silicon wafers in chip lithography can lead to degraded chip performance or even scrappage, making this technology indispensable for achieving nanoscale processing precision. Through the measurement of micro-deformations in materials under stress loading, it is possible to conduct a precise assessment of their mechanical performance, fatigue life and microstructural variations, thereby furnishing essential data support for the research and development of new materials and the optimization of manufacturing processes. In large-scale engineering, micro-displacement sensors detect real-time structural deformations to identify safety risks. Human organ physiological activities are essentially micro-displacement processes, and their high-precision measurement is critical for disease mechanism research, early diagnosis and therapeutic efficacy evaluation.

However, when transferring existing measurement techniques to biomedical applications, several fundamental limitations arise that are frequently overlooked in conventional metrology research. First, traditional measurement methods rely heavily on stable operating conditions and minimal environmental perturbations. In clinical settings, involuntary patient movements, including respiration, muscle tremor, and peristalsis, introduce low-frequency disturbances that compromise the reliability of measurements over extended monitoring periods (Ait et al., 2025). Second, many biomedical phenomena involve displacement amplitudes that span a wide dynamic range. For instance, chest wall motion during respiration, joint range of motion in rehabilitation assessment, and cardiac-induced chest wall pulsation all require measurement systems capable of accommodating both fine resolution (sub-micrometer to micrometer) and large amplitudes (millimeter to centimeter) (Zhao et al., 2022; Wang et al., 2023). This combination of high precision and wide range poses a significant challenge for many existing measurement techniques, which are typically optimized for either small-scale or large-scale displacements but not both simultaneously. Third, most existing systems require manual calibration and operator intervention, limiting their suitability for long-term continuous monitoring in settings such as intensive care units or postoperative recovery rooms. Baseline drift caused by thermal fluctuations, mechanical creep, or gradual laser power decay accumulates over time, compromising accuracy without interrupting the measurement process. Fourth, many interferometric systems are bulky and require direct optical access, conflicting with the requirements of non-contact, unobtrusive monitoring that is increasingly favored in clinical practice to reduce patient discomfort and infection risk.

In existing nanoscale displacement measurement technologies, optical interferometry has gained prominent attention due to its high precision, superior resolution, and traceability to length standards (Zhao et al., 2003; Huang et al., 2025). As a classic experimental instrument in interferometric measurement, the Michelson interferometer translates abstract interference theory into observable physical phenomena. Its well-defined optical path structure and straightforward operating principles have been widely adopted in university physics laboratory teaching. In experiments, when the optical path difference between two arms exceeds the coherent length of the light source, the visibility of interference fringes decreases significantly, which precludes effective measurement. Furthermore, traditional fringe counting methods rely on the manual visual judgment of light–dark fringe variations; these methods not only suffer from low efficiency and poor automation (Luo et al., 2022; Wang et al., 2025), but also exhibit measurement accuracy and reliability that are susceptible to external interference factors such as environmental vibrations and air turbulence (Wang et al., 2021).

To address these issues, this study presents a systematic improvement to the classical Michelson interferometer. The proposed approach, based on Malus’s law, incorporates polarizing optical elements to develop a high-precision mechanical coupling device, which converts the linear micro-displacement of the movable mirror into the angular displacement of the polarizer, thus fundamentally overcoming the limitation of light source coherent length inherent to traditional interferometric measurements. The key insight is that Malus’s law establishes a deterministic, single-valued relationship between polarizer rotation angle and transmitted light intensity ( $I=I_{0}\cos^{2}\theta$ ), where the intensity varies continuously and monotonically within each 90° quadrant without periodic ambiguity. By mechanically coupling the linear displacement of the interferometer’s movable mirror to the angular rotation of a polarizer, the displacement-to-intensity conversion becomes unbounded by coherence length and immune to fringe-ambiguity problems. Furthermore, this approach replaces subjective visual fringe counting with objective photoelectric detection, enabling fully automated, high-resolution displacement measurement suitable for continuous biomedical monitoring. Building on this design, the quantitative relationship between the polarizer’s rotation angle and light intensity is harnessed to map such angular changes to variations in the output light intensity, ultimately enabling the high-sensitivity indirect measurement of micro-displacement.

Building on this physical foundation, the proposed system introduces a hybrid analytic-data-driven framework that unlocks the full precision potential of the hardware design. Unlike conventional approaches that rely on manual calibration or purely empirical compensation, the framework integrates three algorithmic innovations. First, a Particle Swarm Optimization (PSO) algorithm jointly calibrates the mutually coupled hardware parameters—the polarizer initial angle and the photoelectric gain—replacing laborious manual tuning with automated optimization (Ma et al., 2022; Tian et al., 2024). This joint optimization approach explicitly addresses the complex, non-linear interactions between parameters, a limitation that traditional manual or sequential tuning methods fail to overcome. Second, the primary displacement estimate is obtained through analytic inversion of Malus’s law, preserving physical interpretability and computational efficiency. Third, a Gaussian Process Regression (GPR) model compensates residual instrument errors (e.g., gear backlash, laser power fluctuations, thermal drift) beyond the scope of the physical model, while simultaneously providing per-measurement uncertainty bounds. An adaptive online recalibration mechanism further sustains accuracy under long-term drift.

The methodology advances parameter optimization through a specifically adapted PSO, achieving automated, joint calibration of hardware parameters that are typically treated as independent tuning targets. A custom fitness function that simultaneously penalizes measurement uncertainty and computational latency is introduced, representing a novel approach in the context of this analytic inverse mapping problem. This balance between high precision and low latency is crucial for real-time biomedical monitoring applications.

The main contributions of this work are summarized as follows:

1. Principle fusion innovation: A novel integration of Malus’s law with Michelson interferometry is proposed, replacing traditional fringe counting with polarization-based intensity variation, thereby overcoming the coherence-length limitation of the light source on measurement range.
2. Structure and detection innovation: Through gear coupling, linear displacement-to-angle conversion is achieved, replacing manual visual judgment with automated photoelectric detection, completing the paradigm shift from manual counting to photoelectric quantitative measurement.
3. Intelligent processing framework: A hybrid analytic-data-driven framework is developed, comprising PSO-based joint calibration of hardware parameters, analytic inverse mapping for primary displacement computation, and GPR-based residual error compensation with per-measurement uncertainty quantification.
4. Adaptive online recalibration: An adaptive online recalibration mechanism is introduced to sustain accuracy under long-term drift, enabling continuous monitoring applications.
5. Biomedical application exploration: The application potential in biomedical micro-displacement monitoring is systematically explored, providing a feasible technical pathway for the development of novel diagnostic and therapeutic devices.

Several key innovations are introduced in this work. A polarization modulation unit is incorporated into the optical path, which eliminates the dependence on interference fringe visibility and theoretically extends the measurement range without coherence-length constraints. To address the difficulty of manually calibrating hardware parameters, PSO is employed for joint calibration of the polarizer initial angle and the photoelectric amplifier gain, ensuring that the system consistently operates in the most sensitive region. Additionally, GPR is adopted to compensate for residual instrument errors that the physical model cannot capture, such as gear backlash and laser power fluctuations, while also providing per-measurement uncertainty bounds. Together, these innovations offer a novel technical solution for high-precision, wide-range micro-displacement measurement.

## 2 Principle

### 2.1 Michelson interferometer and its limitations

**Figure 1:**
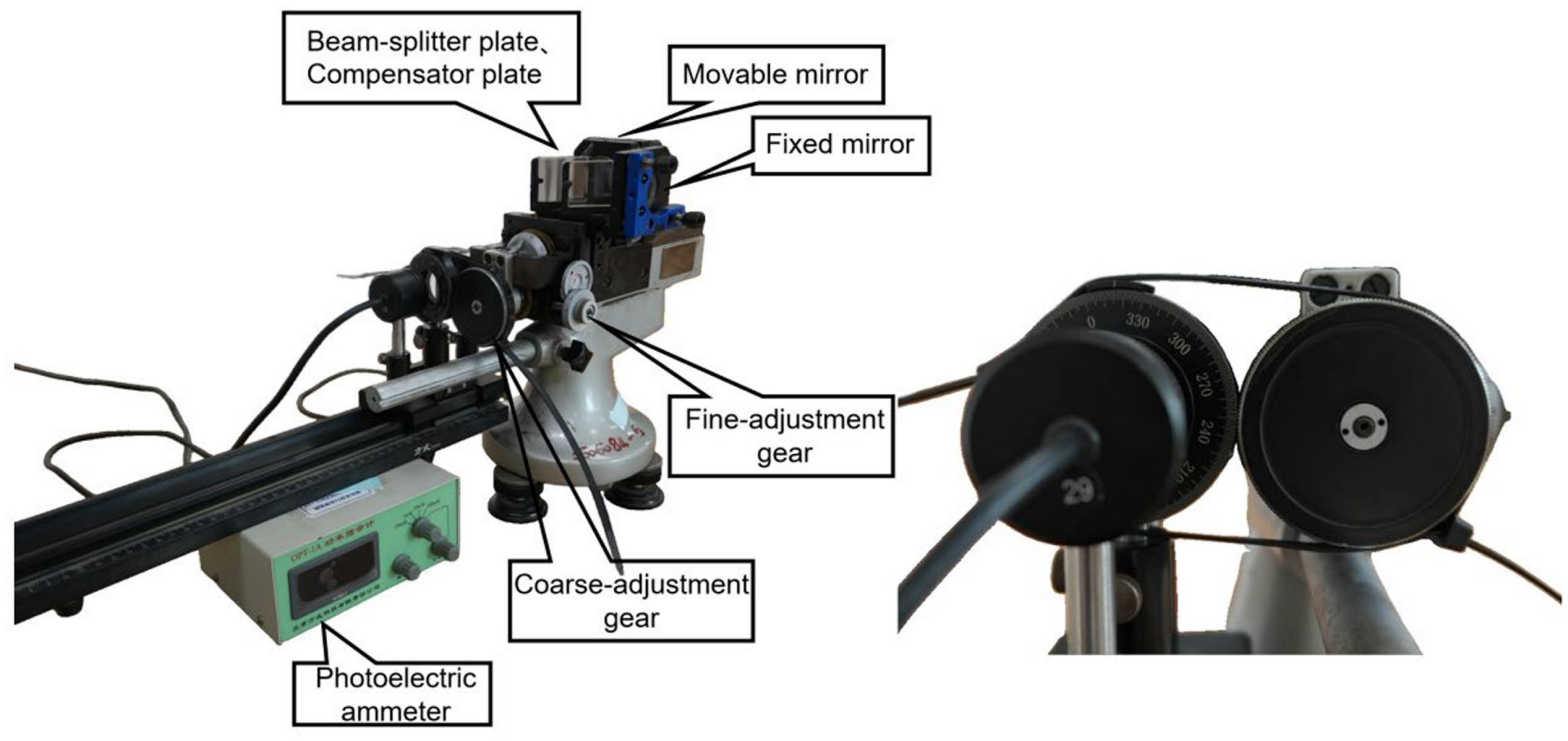
Improved Michelson interferometer device diagram and gear coupling diagram.

The Michelson interferometer is a classic optical instrument that adopts the amplitude-splitting method to produce double-beam interference (Qiao et al., 2015). The configuration of the Michelson interferometer commonly used in laboratory settings is illustrated in Figure 1, which primarily comprises a laser, a beam splitter (G1), a compensation plate (G2), a movable mirror (M1), a fixed mirror (M2), a guide rail, and adjustment screws.

Rotation of the coarse and fine handwheels drives the translational movement of the movable mirror (M1) along the guide rail, which in turn modulates the optical path difference between the two light beams and induces the shift of interference fringes. The displacement Δd of the moving mirror and the fringe change Δk satisfy the following relationship (Equation 1):

$$\mathrm{\Delta d}=\mathrm{\Delta k}\cdot (\frac{\lambda }{2})$$ (1)

where *λ* denotes the laser wavelength. The equation demonstrates that each fringe shift within the interference field corresponds to a displacement of λ/2 of the movable mirror. However, traditional fringe-counting methods exhibit inherent limitations. First, their effective measurement range is constrained by the coherent length of the laser source. When the optical path difference between the two arms exceeds this length, fringe visibility degrades significantly, precluding further measurements (Liao et al., 2024). Secondly, such methods rely on the manual visual judgment of fringe shifts, which is not only inefficient but also susceptible to measurement inaccuracies induced by environmental vibrations, air turbulence, and subjective judgment errors of the operator.

### 2.2 Malus’s law

When linearly polarized light passes through a polarizer, the relationship between the transmitted light intensity I_2_ and the incident light intensity I_1_ follows Malus’s law, expressed as (Equation 2):

$$I_{2}=I_{1}\cdot \cos^{2}\alpha$$ (2)

where *α* denotes the angle between the vibration direction of the linearly polarized light and the transmission axis of the analyzer. Physically, this relationship arises because a polarizer only transmits the electric field component parallel to its transmission axis, and the light intensity is proportional to the square of the amplitude. Malus’s law thus provides a precise mathematical foundation for converting angular variations into measurable intensity changes, which is central to the proposed measurement scheme.

**Figure 2:**
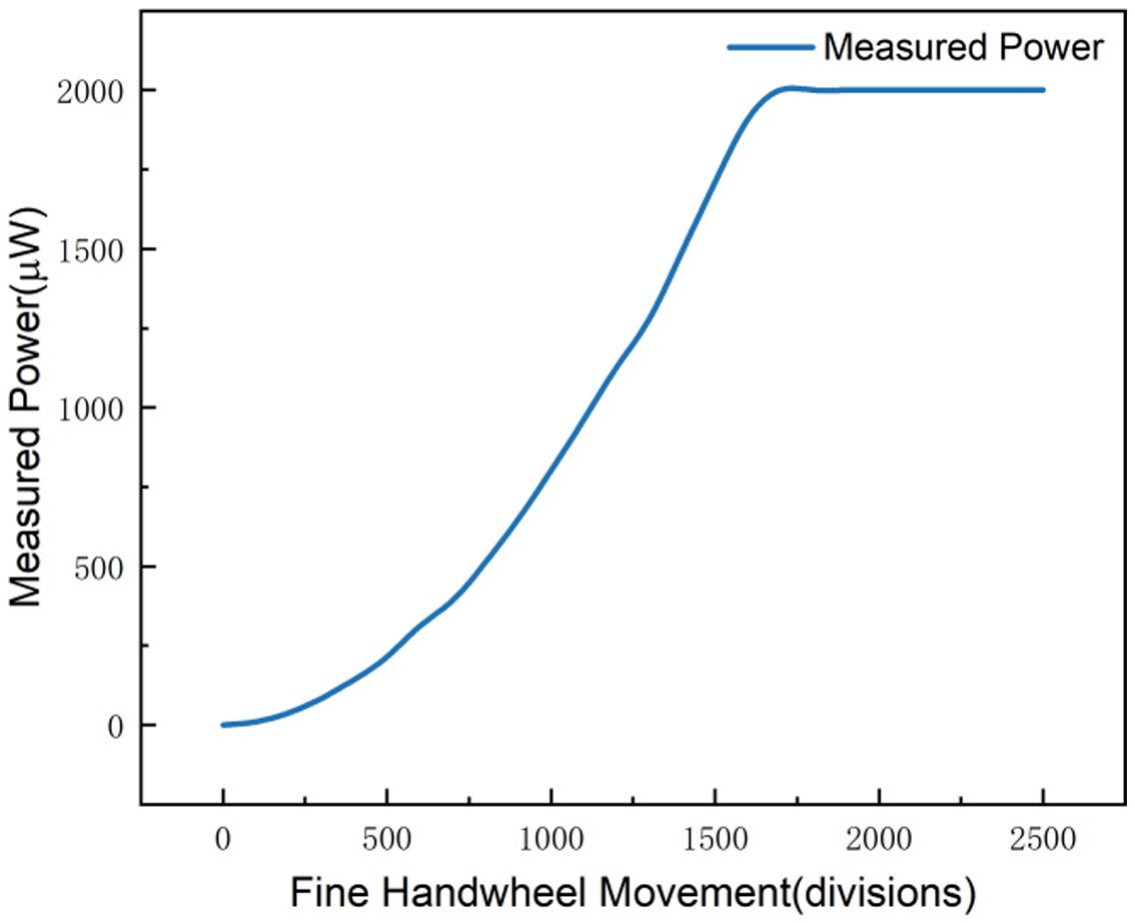
Data out of range.

Malus’s law arises from the vector nature of electromagnetic waves. When linearly polarized light with electric field amplitude $E_{0}$ encounters an analyzer, only the component of the electric field parallel to the transmission axis can pass through. This transmitted component has amplitude $E=E_{0}\text{cosα}$ (Figure 2). Since light intensity is proportional to the square of the electric field amplitude ( $I\propto E^{2}$ ), the transmitted intensity becomes:

$$\begin{array}{c} I_{2}=I_{1}\cos^{2}\alpha =I_{1}\cdot \frac{E^{2}}{E_{0}^{2}}=I_{1}\cdot \frac{{\left(E_{0}\text{cosα}\right)}^{2}}{E_{0}^{2}}=I_{1}\cos^{2}\alpha \end{array}$$ (3)

In the context of this study, the linear displacement *x* of the movable mirror is mechanically coupled to the rotation angle *θ* of the polarizer through a gear transmission system with coupling ratio k, such that:

$$\begin{array}{c} \theta =\theta_{0}+\mathrm{kx} \end{array}$$ (4)

where θ₀ is the initial angle of the polarizer. Substituting Equation 4 into Equation 3, the detected light intensity p can be expressed as a function of displacement x (Equation 5):

$$\begin{array}{c} p=G\cdot I_{\max}\cdot \cos2(\theta_{0}+\mathrm{kx}) \end{array}$$ (5)

where G is the gain setting of the photoelectric ammeter, and I_max_ is the maximum incident light intensity. This equation establishes the quantitative relationship between displacement and light intensity, forming the physical foundation for the proposed measurement system.

The sensitivity of the system, defined as the rate of change of intensity with respect to displacement, is given by:

$$\begin{array}{c} \begin{array}{l} \partial p/\partial x=-2\mathrm{Gk}I_{\max}\cdot \cos(\theta_{0}+\mathrm{kx})\cdot \sin(\theta_{0}+\mathrm{kx})=\\ -\mathrm{Gk}I_{\max}\cdot \sin(2\theta_{0}+2\mathrm{kx}) \end{array} \end{array}$$ (6)

Equation 6 reveals that maximum sensitivity is achieved when *θ*₀ + kx = 45 ° (or odd multiples thereof), where the slope of the cos^2^ curve is steepest. This insight guides the PSO-based calibration to position the operating point at the maximum-slope region.

## 3 Experimental apparatus and design

### 3.1 Experimental instruments

Optical components such as Michelson interferometer, helium-neon laser, polarizer, optical anti-vibration platform, space filter, beam expander, collimator, and electronic equipment such as power supply and photoelectric current measuring instrument.

### 3.2 Experimental instruments

To overcome the limitations in measurement accuracy of the traditional Michelson interferometer, this study proposes an innovative modification strategy based on Malus’s law. As illustrated in Figure 1, the proposed scheme integrates a polarization assembly into one arm of the optical path of the Michelson interferometer, with the micrometer handwheel of the interferometer mechanically coupled to the polarizer. Rotation of the micrometer handwheel drives the movable mirror to generate a linear displacement, whereby the displacement Δd is linearly converted into an angular variation Δθ of the polarizer. According to Malus’s law, this angular variation induces a change in the transmitted light intensity. By detecting variations in the output light intensity signal via a photoelectric ammeter, the system enables high-sensitivity indirect measurement of linear displacement.

During the experimental setup, the structure of the polarizing optical assembly was optimized to satisfy the spatial requirements for mechanical coupling. In the system calibration phase, the polarizer was fixed at the laser output port to maintain a constant orientation of its transmission axis, thereby providing linearly polarized light with a stable vibration direction.

**Figure 3:**
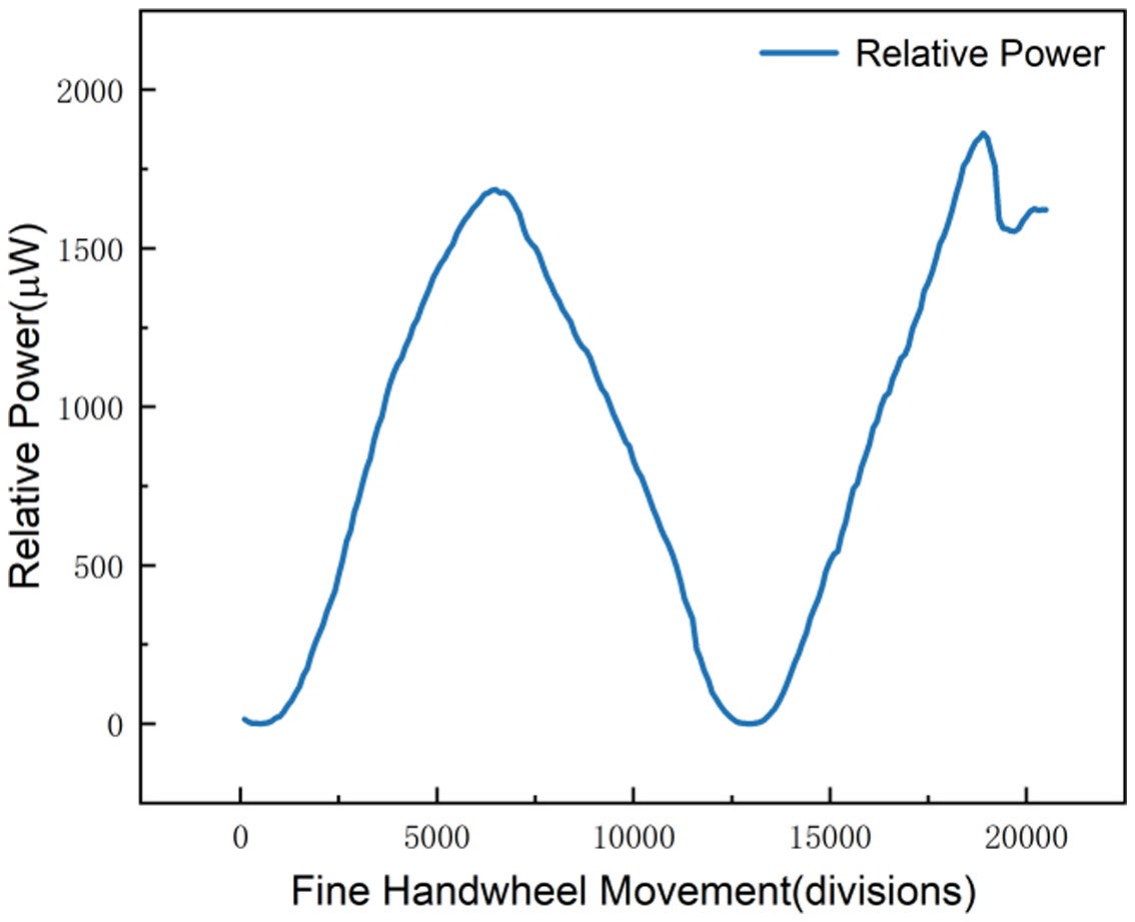
Instrument smoothness.

As illustrated in Figure 2, preliminary tests indicated that the readings of the photoelectric ammeter exceeded its measurement range under high-precision operating conditions (full scale: 2000 units). To avoid such range exceeding, the polarizer was fixed at a fixed angle relative to the laser output port to ensure a stable light transmittance, while the zero point and measurement range of the ammeter were adjusted to keep the output signal within its operational limits. Subsequent experiments (Figure 3) revealed that loose gear meshing led to thread slippage, which caused anomalous fluctuations in the measured data. To address this issue, the relevant components of the device were secured with cable ties, ensuring the accuracy and reliability of all subsequent measurements. This design eliminates the dependence on interference fringe visibility, which theoretically enables the infinite extension of the measurement range.

## 4 Experimental procedure

### 4.1 Instrument adjustment

- Switch on the laser power supply;
- Adjust the optical components on the guide rail to ensure the optical axis is horizontal, such that the photoelectric ammeter can receive a uniform bright spot;
- Couple and fix the polarizer gear with the coarse adjustment gear of the Michelson interferometer.

### 4.2 Adjusting instrument parameters

- Set the photoelectric ammeter to its maximum accuracy, then rotate the zero-adjustment knob to minimize the current reading;
- Coarsely adjust the polarizer: cease adjustment when the light intensity reading reaches its maximum value, then re-adjust the zero-adjustment knob to calibrate the maximum reading within the range of 1900–1950 units, which corresponds to the maximum allowable measurement accuracy of the instrument.

### 4.3 Operation step

#### 4.3.1 Preliminary testing experiment

- Rotate the micrometer gear one full revolution to observe a rotational displacement of approximately 1.5–2 ° of the polarizer. Once the light intensity reading stabilizes, record the corresponding value, and repeat this procedure until the polarizer completes a full 360 ° rotation.

#### 4.3.2 Precision testing experiment

- Rotate the polarizer continuously and observe the periodic fluctuations in the light intensity readings during a full 360 ° rotation, then record the maximum and minimum values obtained;
- Adjust the micrometer gear to measure the micro-displacement within a range of 100 units, which corresponds to half the absolute range of the light intensity-displacement (p-x) curve.

## 5 Experimental results and discussion

### 5.1 Experimental data processing

#### 5.1.1 Preliminary testing experiment

**Figure 4:**
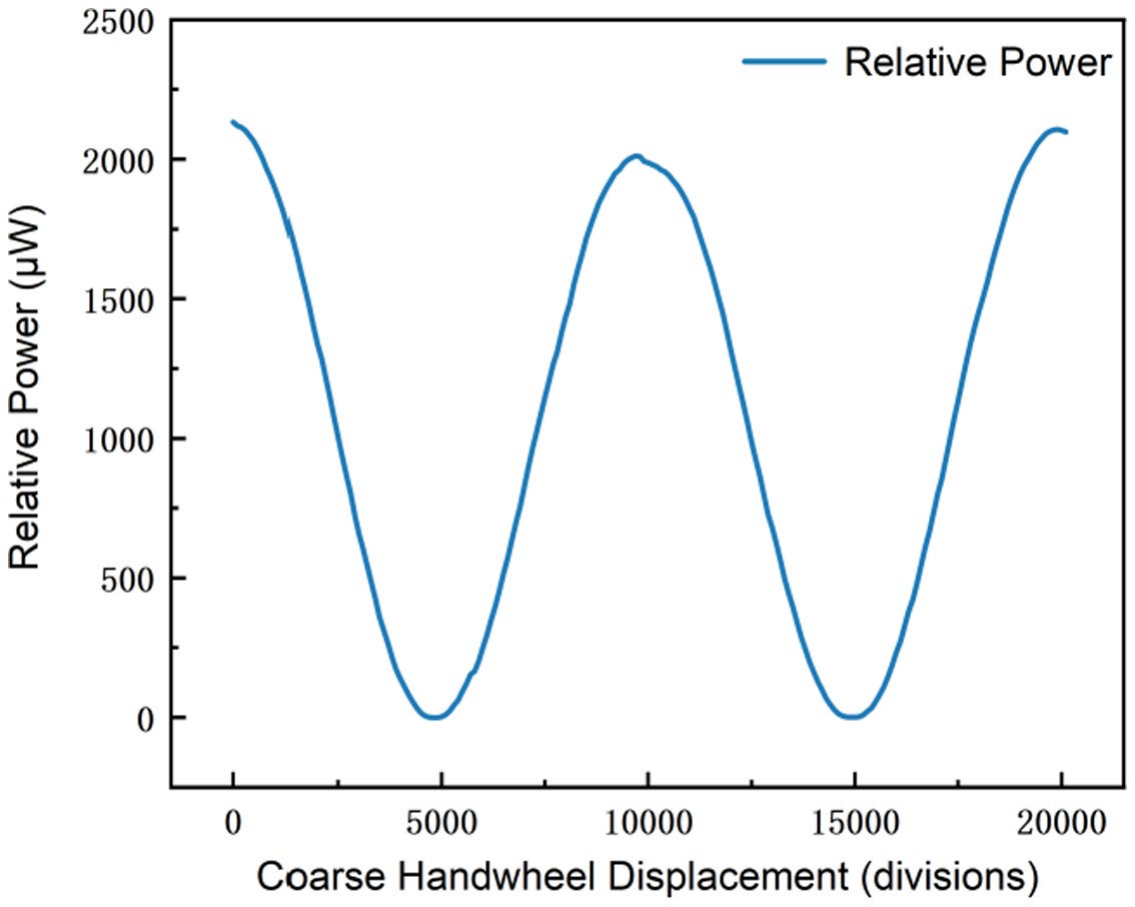
Experimental image of rough measurement.

Experimental data indicate that, as the polarizer completes a full rotational cycle (Figure 4), the readings on the display exhibit periodic variations, with the maximum and minimum values within each cycle remaining stably within the respective defined ranges. When the experimental data are plotted in a coordinate system with the display reading p as the y-axis and the displacement x as the x-axis, the resulting curve presents a quasi-cosine profile, which is consistent with Malus’s law that describes the relationship between the polarizer rotation angle *θ* and the output light intensity p. When plotted with the light intensity p as the y-axis and the polarizer rotation angle θ as the x-axis, the curve exhibits a perfect cosine-squared profile. Since the displacement x drives the polarizer to rotate linearly via gear coupling, small-range measurements performed at the point of maximum slope on the cos^2^θ curve (where $\theta \approx 45^{{}^{\circ}}$ ) allow this nonlinear relationship to be approximated as a linear one, thereby enabling the high-sensitivity linear conversion between displacement x and light intensity p.

#### 5.1.2 Precision testing experiment

- For precise measurements at higher slope positions

**Figure 5:**
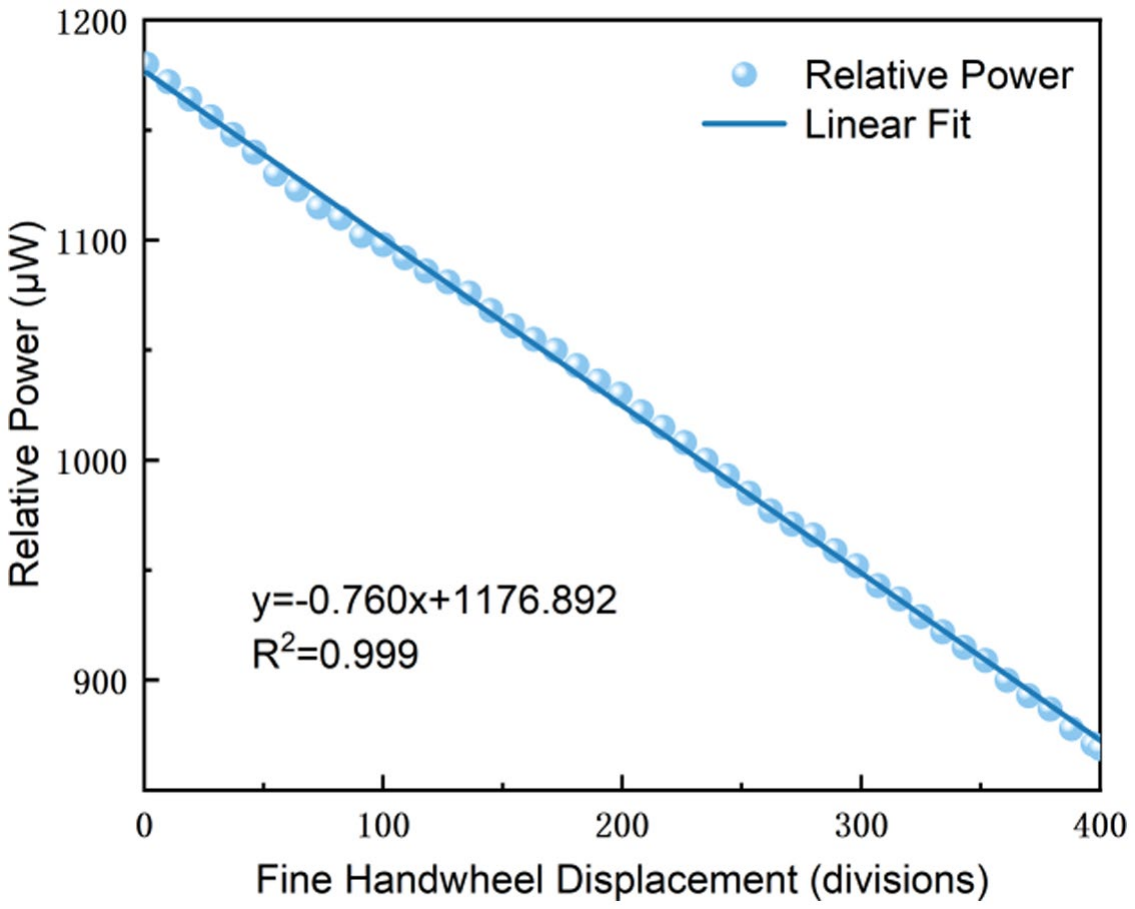
Experimental image of fine measurement with high slope.

**Figure 6:**
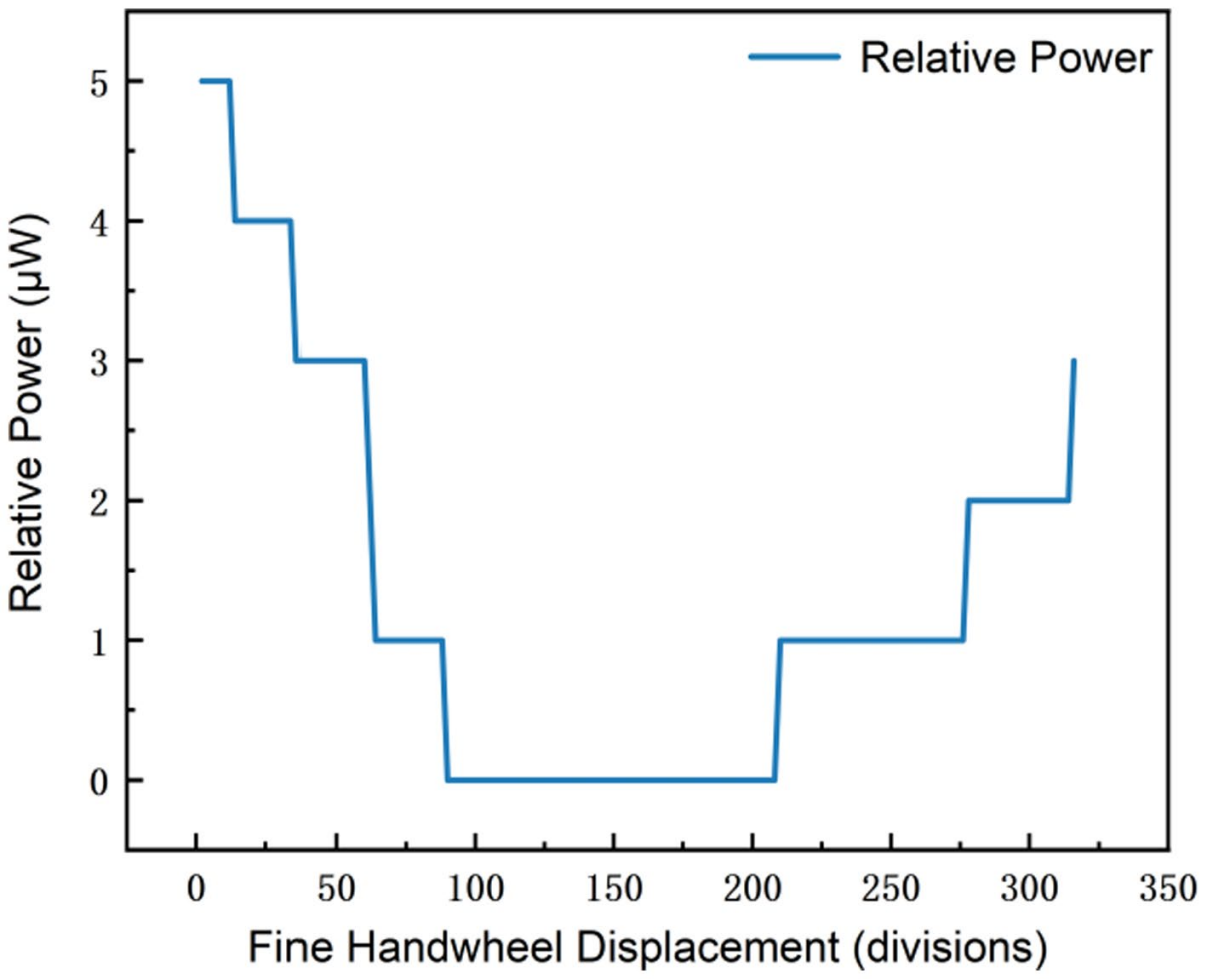
Experimental image of fine measurement with low slope.

Experimental data indicate that within this range, a single notch rotation of the Michelson interferometer’s micrometer handwheel per cycle elicits a linear variation in the polarizer-associated display reading, which ensures a consistent 1-unit reading change per notch rotation. The data fitting results presented in Figure 5 yield a linear regression equation with a coefficient of determination *R*^2^ = 0.999, which demonstrates a high degree of model fitting and a strong linear correlation between the measured variables;

- For precise measurements at lower slope positions:

Experimental data (Figure 6) show that within this range, the polarizer’s display value changes by only 1 after multiple rotations when the micrometer handwheel of the Michelson interferometer is turned two stops;

- Michelson interference experiment:

The traditional Michelson interference experiment data are shown in Table 1, which is used to compare the precision with the improved system.

**Table 1:** Michelson interference experiment data.

Serial no.	Position reading *d*/mm	Position reading *d’*/mm	*d*-*d’*/mm
1	64.32148	64.33739	0.01591
2	64.35212	64.36818	0.01606
3	64.36818	64.38431	0.01613
4	64.38431	64.40031	0.01600
5	64.41769	64.43367	0.01598
6	64.43367	64.44959	0.01592
7	64.44959	64.46560	0.01601
8	64.46560	64.48142	0.01582
9	64.48142	64.49750	0.01608
10	64.52968	64.54559	0.01591

### 5.2 Data analysis

#### 5.2.1 Precision testing experiment

- Precision test 1, signal change per notch at the steep gradient:

∆y/∆x = 0.777,

Within the quasi-linear operating range, the system resolves a displacement of approximately 1.287 notch units (0.0001287 mm) for a 1-unit variation in light intensity. This result directly demonstrates the enhanced measurement precision and high-resolution performance of the modified instrument;

- Precision test 2, signal change per notch at the steep gradient:

∆y/∆x = 0.059,

Within the nonlinear operating range, the system resolves a displacement of 17 notch units (0.0017 mm) for a 1-unit variation in light intensity. Data obtained at the gentle gradient serve only as a reference and do not reflect the enhanced measurement precision of the modified instrument;

- Theoretical displacement per notch for the conventional Michelson interferometer:

∆d = *λ*∆k/2 = 0.0003165 (λ = 633 nm).

For the conventional measurement system, the displacement corresponding to one resolution unit (i.e., the minimum detectable variation of traditional methods) is 3.165 notch units, equivalent to 0.0003165 mm.

It can be seen from the experimental results that the improvement in measurement accuracy is mainly attributed to the polarization modulation mechanism itself. By converting linear displacement into a continuous light intensity signal through Malus’s law, the system bypasses the manual fringe counting step used in conventional methods, thereby eliminating the subjective errors inherent to that approach. In the region of maximum slope on the cosine-squared curve (around $\theta \approx 45^{{}^{\circ}}$ ), the light intensity is most sensitive to angular changes, allowing the system to resolve displacement increments smaller than those detectable by a conventional interferometer. In our tests, a resolution of 0.13 μm was achieved. In contrast, near the peaks or troughs of the curve ( $\theta \approx 0^{{}^{\circ}}\;\text{or}\;90^{{}^{\circ}}$ ), the slope approaches zero, and the same angular rotation produces only a weak change in intensity, leading to noticeably larger measurement errors. This suggests that in practice, the choice of initial angle is critical. To make the best use of this setup, the operating point should be set within the steep-slope region, or alternatively, tracking of the operating point should be implemented during measurements.

In terms of repeatability, three independent trials were conducted under identical conditions. With the micrometer position fixed in the steep-slope region, the standard deviation of the displacement readings was approximately 0.11 μm, indicating good short-term stability. However, during the experiments it was observed that the intensity baseline drifted slowly over extended operation. This is likely due to thermal expansion of the mechanical components, and the gear coupling itself may also be affected by temperature changes. Proactively recalibrating the zero point every 30 min helped to suppress this drift and maintain measurement reliability.

When compared to a conventional Michelson interferometer, the advantage of this modified system lies not only in the higher measurement accuracy achieved in the steep-slope region, but also in the fact that the measurement range is no longer limited by the coherence length of the laser. In conventional experiments, once the optical path difference exceeds the coherence length, the interference fringes become blurred or even disappear. The system proposed here operates on the principle of intensity modulation, so in theory the measurable range is determined by the mechanical travel of the setup. This feature is especially valuable in applications that require a large dynamic range, such as long-term monitoring of slow structural deformation or continuous tracking of joint motion angles.

Overall, the experimental results demonstrate that the approach of converting displacement into a polarizer rotation angle via mechanical coupling and then reading the intensity via Malus’s law can effectively enhance the measurement capability of a Michelson interferometer. The trade-offs between sensitivity and measurement range, as well as the issue of long-term stability identified in this analysis, provide a basis for incorporating active calibration and error compensation strategies in future work.

#### 5.2.2 Uncertainty calculation

- Relative uncertainty of the rotational variable

Relative uncertainty of the rotational variable for the relative power in preliminary tests = 8.1%.

Relative uncertainty of the rotational variable at the steep gradient in precision test 1 = 9.0%.

Relative uncertainty of the rotational variable at the gentle gradient in precision test 2 = 5.7%;

- Relative uncertainty of relative power

Relative uncertainty of relative power in preliminary tests = 1.1%.

Relative uncertainty of relative power at the steep gradient in precision test 1 = 0.9%.

Relative uncertainty of relative power at the gentle gradient in precision test 2 = 0.05%;

- Intrinsic uncertainty of the Michelson interferometer

*μ*= $\sqrt{2}$ ∆Instrument = 0.00007.

In summary, the modified device exhibits the following operational characteristics: within the steep-gradient regions of the Malus’s law curve, its measurement accuracy exceeds the intrinsic precision of the conventional Michelson interferometer; conversely, within the gentle-gradient regions, its accuracy is inferior to that of the conventional interferometer. The overall improvements of the device are manifested in two key aspects. First, the measurement system achieves a 2.46-fold improvement in precision relative to traditional methods within the high-accuracy measurement range. Second, by adopting the periodic measurement principle based on polarization modulation, the device eliminates the dependence on visible interference fringes, thereby overcoming the measurement limitations imposed by the coherent length of the light source. Under ideal conditions, its measurable range extends from 1.287 μm to infinity; in contrast, the conventional Michelson interferometer has a limited effective measurement range due to coherence constraints. Consequently, the modified device inherently possesses a substantially broader measurable range than the traditional Michelson interferometer.

### 5.3 Medical application prospects

Biomedical signals carry critical information that reflects the physiological states and pathological changes of the human body. Their effective acquisition and accurate analysis play an irreplaceable role in clinical diagnosis, treatment evaluation, and health monitoring (Li et al., 2010). With the continuous advancement of medical intelligence, biomedical signal processing is moving toward the development of high-precision, non-contact, and long-term monitoring technologies. Against this backdrop, the modified measurement device developed in this study exhibits broad application prospects in diverse clinical scenarios by virtue of its advantages of high sensitivity, non-invasiveness, and a wide dynamic range.

#### 5.3.1 Joint range of motion monitoring

- Motor rehabilitation assessment

A micro-transmission device is attached to articular joints (e.g., the knee and elbow) to capture real-time micro-displacements (micron scale) during flexion movements, thus quantifying the joint range of motion and muscle coordination performance. For example, in rehabilitation training, a micro-rotary device is affixed to the joint surface, and the transmission mechanism is utilized to conduct real-time monitoring of rotational variables, enabling the objective evaluation of motor function rehabilitation outcomes. This method is applicable for the follow-up rehabilitation of patients with stroke or spinal cord injury, achieving a measurement precision of 0.1° for angular variations.

- Prosthetic fitting optimization

Optical fiber sensors are integrated at the contact interface between a prosthesis and the residual limb to detect micron-scale displacement variations, which in turn optimizes the interfacial pressure distribution and reduces skin abrasion at the contact site.

- Dynamic stability analysis

The experimental setup is modified to capture instantaneous displacements during high-speed joint movements, and the acquired data can be applied to the prevention of sports-related injuries in athletes.

#### 5.3.2 Thoracoabdominal displacement measurement

- Respiratory monitoring

a. Thoracic wall movement detection

A white-light interferometer is used to measure the periodic displacement of the thoracic wall during respiration (approximately 0.5–2 mm), enabling the analysis of respiratory rate, rhythm, and depth. This method is suitable for long-term monitoring of patients with chronic obstructive pulmonary disease (COPD) and can identify abnormal respiratory patterns with a displacement deviation as small as 0.05 mm.

b. Non-invasive pulmonary function assessment

Polarization spectroscopy technology is integrated to eliminate interference from skin surface reflection, allowing for the accurate detection of thoracic cavity displacement during pulmonary expansion and contraction. This approach provides a viable alternative to traditional spirometers for pulmonary function assessment.

c. Sleep apnea diagnosis

Non-contact monitoring of nocturnal thoracoabdominal movements is achieved, which eliminates the discomfort caused by traditional chest band sensors and enables continuous overnight respiratory monitoring with a detection sensitivity of 0.01 mm.

- Cardiac pulsation monitoring

a. Apical pulsation displacement measurement

The high-sensitivity modified instrument is utilized to detect nanoscale vibrations of the precordial thoracic wall induced by cardiac pulsation. This measurement enables the evaluation of cardiac pumping efficiency, myocardial contractility, and cardiac output, thereby assisting in the clinical diagnosis of heart failure. The instrument achieves a sampling rate of up to 1 kHz, which allows for the accurate capture of electromechanical delay in cardiac activity.

b. Vascular wall elasticity analysis

The vascular elastic modulus is retroactively calculated from the displacement of the thoracic aorta derived from measurements. This method facilitates the early screening of arteriosclerosis with a measurement accuracy of 5 nm.

c. Postoperative monitoring

Non-contact monitoring of heart rate variability in postoperative patients is implemented, which avoids the risks of skin allergy and infection associated with the use of traditional electrode patches.

#### 5.3.3 Technical advantages

- *Non-invasiveness*: The device eliminates the requirement for direct contact with surface electrodes or sensors, thereby minimizing the risk of infection and improving patient comfort during monitoring.
- *Electromagnetic interference immunity*: Optical-based measurement technology is not affected by electromagnetic interference from medical equipment such as magnetic resonance imaging (MRI) scanners and high-frequency electrosurgical units.
- *Long-term stability*: It exhibits no baseline drift, making it suitable for continuous monitoring in intensive care units (ICU) for periods exceeding 72 h.
- Multi-parameter synchronization: A single device is capable of simultaneously measuring displacement, frequency, and phase information, enabling integrated data acquisition.

## 6 System design

### 6.1 Motivation and overview

The improved interferometer described in section 2 replaces manual fringe counting with a continuous light intensity signal p produced by the polarizer-coupled photoelectric ammeter. Since the relationship between the detected intensity p and the mirror displacement x is fully described by the analytic expression of Malus’s law, displacement is recovered through direct analytic inversion as the primary computational pathway.

**Figure 7:**
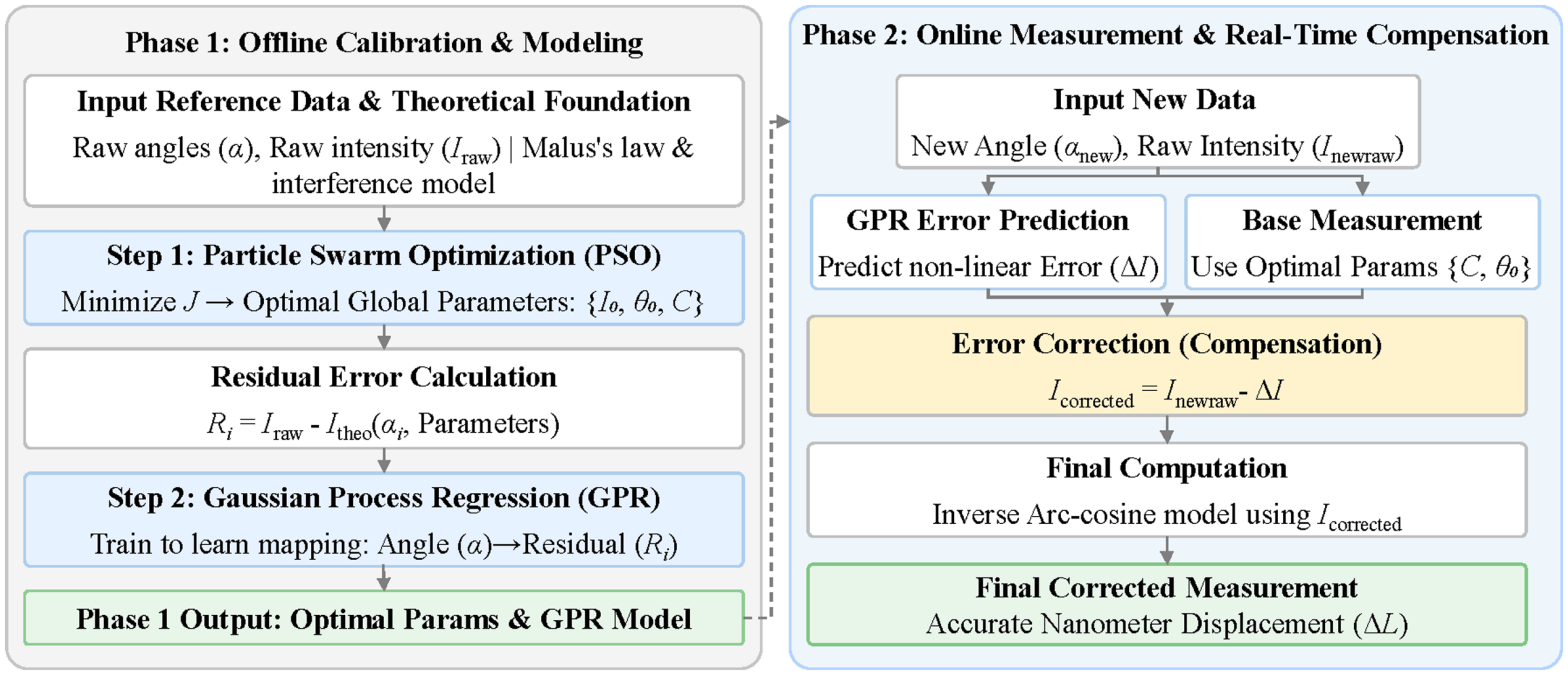
Overall framework.

However, realizing the full precision potential of this analytic pathway requires addressing two practical challenges that the physical model alone cannot resolve. First, the accuracy of the analytic inverse mapping depends critically on the calibration of two hardware parameters—the polarizer initial angle $\theta_{0}$ and the gain setting G of the photoelectric ammeter—which cannot be set optimally by manual adjustment alone. Second, real instruments inevitably exhibit residual errors that lie outside the scope of the Malus’s law model, including backlash in the gear coupling, non-uniform laser power, and slow thermal drift of optical components; these errors persist even after perfect analytic inversion and must be compensated separately. This section proposes three algorithmic modules to address these challenges: As illustrated in Figure 7, a Particle Swarm Optimization (PSO) (Shami et al., 2022; Piotrowski et al., 2023; Tijjani et al., 2024) algorithm for joint calibration of $\left(\theta_{0},G\right)$ , an analytic inverse mapping module as the primary displacement computation path, and a Gaussian Process Regression (GPR) (Aigrain and Foreman-Mackey, 2023; Jin and Xu, 2024; Ma et al., 2024) model dedicated exclusively to compensating the residual instrument errors that the analytic model cannot capture. An adaptive online recalibration mechanism is further introduced to sustain accuracy under long-term drift. The role of PSO in this design is threefold: (1) it automates the calibration process that would otherwise require laborious manual tuning; (2) it performs joint optimization of the two interdependent parameters (*θ*₀ and G), accounting for their non-linear interactions; and (3) it balances the trade-off between measurement precision and computational latency through a custom fitness function, ensuring real-time applicability.

While several intelligent optimization algorithms exist, including Genetic Algorithm (GA), Ant Colony Optimization (ACO), and Differential Evolution (DE), PSO was selected for this application based on the following considerations: PSO requires fewer function evaluations compared to GA, which involves expensive genetic operations (crossover, mutation) and larger population sizes. For real-time calibration requiring latency below 2 ms, PSO’s lower computational overhead is advantageous. The calibration parameters (*θ*₀ and G) are continuous variables. PSO naturally handles continuous optimization problems, whereas ACO is primarily designed for discrete combinatorial problems and requires discretization adaptations. PSO has fewer hyperparameters to tune (primarily w, c₁, c₂) compared to GA (population size, crossover rate, mutation rate, selection strategy), making it easier to implement and less sensitive to parameter settings. For the low-dimensional (2D) search space in this problem, PSO exhibits rapid convergence (within 50 iterations) without premature convergence to local optima, as demonstrated in our experiments. PSO only stores particle positions and velocities, requiring O(N·D) memory where N is swarm size and D is dimensionality. This is more memory-efficient than GA’s population storage and DE’s mutation vector storage. PSO has been successfully applied in numerous optical system calibration problems, including laser parameter optimization and sensor calibration, demonstrating its suitability for this domain.

Based on these considerations, PSO provides the optimal balance of calibration accuracy, computational efficiency, and implementation simplicity for the joint hardware parameter calibration task in this study.

The processing pipeline is intentionally divided into offline calibration and online measurement phases to balance calibration accuracy with real-time performance. In the offline phase, PSO performs extensive global search (50 iterations) to determine optimal hardware parameters (*θ*₀*, G*), while comprehensive reference measurements are collected to train the GPR residual error model and optimize kernel hyperparameters—operations that are computationally intensive but unconstrained by time. In the online phase, these pre-calibrated parameters and the trained GPR model enable real-time displacement measurement through efficient analytic inverse mapping and single matrix–vector multiplication for residual correction, with strict latency requirements (<2 ms per measurement). The offline phase thus establishes the “knowledge base” that empowers efficient online operation, while the online phase continuously monitors residual statistics and GPR uncertainty to detect drift. When necessary, incremental recalibration (local PSO search of 10 iterations and GPR update with 50 new points) is triggered without interrupting the measurement stream, ensuring sustained accuracy throughout long-term monitoring.

### 6.2 Analytic inverse mapping as the primary displacement computation path

Since the polarizer rotation angle θ is linearly related to the mirror displacement x through the gear coupling ratio k, that is $\theta =\mathrm{kx}+\theta_{0}$ , and the detected intensity follows Malus’s law $p=I_{0}\cos^{2}(\mathrm{kx}+\theta_{0})$ , the displacement can be recovered analytically from a measured intensity value p as (Equation 7):

$$\begin{array}{c} x_{\text{analytic}}=\frac{\arccos(\sqrt{\frac{p}{I_{0}}})-\theta_{0}}{k} \end{array}$$ (7)

where $I_{0}$ is the maximum intensity recorded during calibration. This closed-form expression requires no training data and introduces no approximation error from the physical model itself. It is evaluated in real time on the microcontroller unit (MCU) and constitutes the primary output of the measurement pipeline. The accuracy of $x_{\text{analytic}}$ depends directly on the precision of the calibrated parameters $\theta_{0}$ and k , which motivates the PSO-based calibration module described in subsection 3.3.

### 6.3 PSO-based joint calibration of hardware parameters

The precision of the analytic inverse mapping is sensitive to two hardware-level parameters: the polarizer initial angle $\theta_{0}$ and the gain setting G of the photoelectric ammeter. An error in $\theta_{0}$ shifts the operating point away from the maximum-slope region of the $\cos^{2}$ curve, reducing sensitivity; an inappropriate G causes signal saturation or excessive quantization noise, both of which degrade measurement resolution. Because these two parameters interact—a change in G alters the effective dynamic range within which $\theta_{0}$ should be positioned—they must be optimized jointly rather than sequentially. Manual tuning of this two-dimensional configuration space is laborious and prone to suboptimal solutions, motivating the use of a Particle Swarm Optimization (PSO) algorithm to perform the joint calibration automatically.

**Figure 8:**
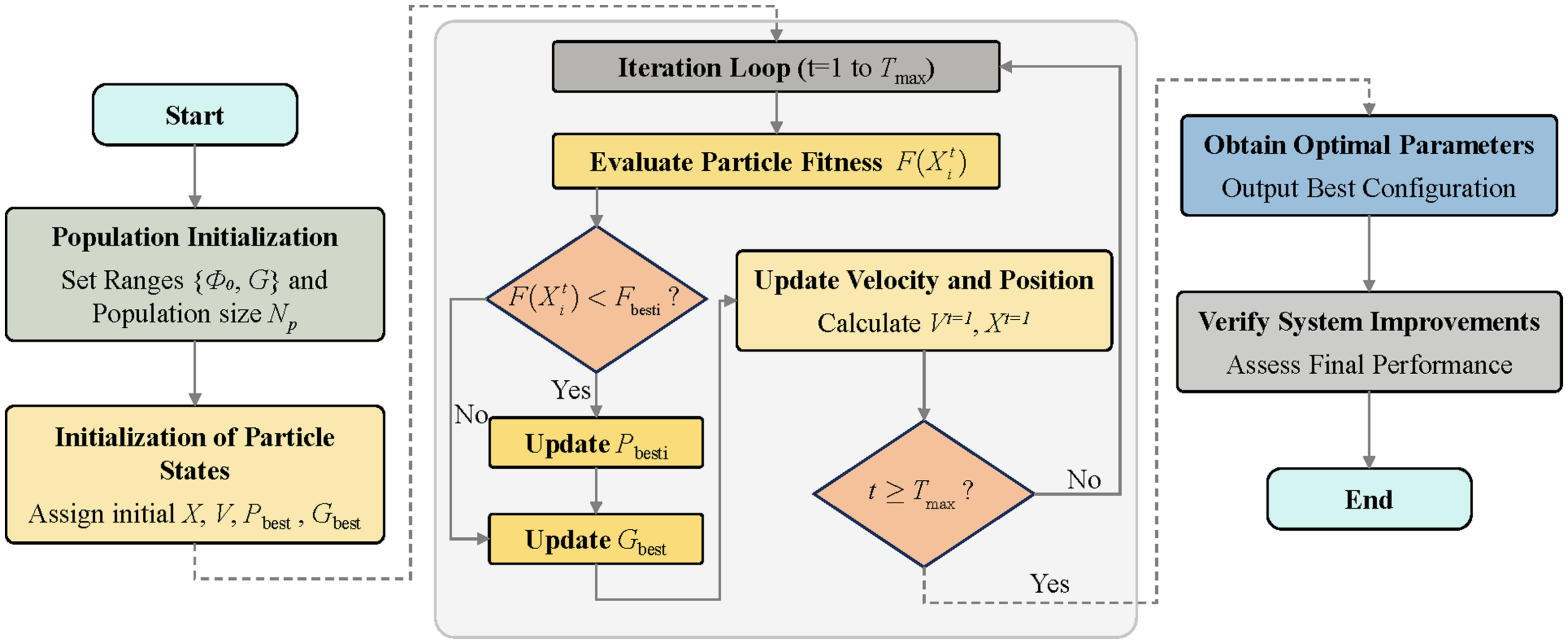
Flowchart for PSO-based joint hardware parameter calibration.

PSO is a population-based stochastic optimization technique inspired by the social behavior of bird flocking and fish schooling. The algorithm models the search space as a conceptual “flight field,” in which each particle (representing a potential solution) adjusts its flight path based on its personal best experience ( $p_{\text{best}}$ ) and the collective best experience of the entire swarm ( $g_{\text{best}}$ ), thereby exploring the space to find optimal solutions. The detailed flowchart of the proposed PSO-based joint calibration of hardware parameters is shown in Figure 8 (Tian et al., 2024).

The PSO operates on a swarm of N=30 particles over a two-dimensional search space with $\theta_{0}$ in [30°, 60°] and G in (Ait et al., 2025; Tian et al., 2024). Each particle i maintains a position vector ( $v_{i}={\left(\theta_{0},G\right)}_{i}$ and a velocity vector $u_{i}$ , updated at each iteration t according to (Equations 8,9):

$$\begin{array}{c} u_{i}(t+1)=\omega u_{i}(t)+c_{1}r_{1}(p_{\text{best},i}-v_{i}(t))+c_{2}r_{2}(g_{\text{best}}-v_{i}(t)) \end{array}$$ (8)

$$\begin{array}{c} v_{i}(t+1)=v_{i}(t)+u_{i}(t+1) \end{array}$$ (9)

where ω=0.729 , $c_{1}=c_{2}=1.494$ , and $r_{1},r_{2}$ are independent random scalars drawn from U(0,1) . The objective of the PSO search is to find the optimal combination of hardware parameters (*θ*₀*, G*) that minimizes the following fitness function (Equation 10):

$$\begin{array}{c} f(\theta_{0},G)=w₁\cdot \sigma_{\text{measurement}}(\theta_{0},G)+w₂\cdot t_{\text{latency}}(\theta_{0},G) \end{array}$$ (10)

where σ_measurement_(θ₀, G) is the measurement uncertainty under parameters (θ₀, G), calculated as the standard deviation of residuals between analytically computed displacement and reference standard measurements. t_latency_(θ₀, G) is the computational latency for processing a single measurement, including analytic inversion and GPR correction. w₁ and w₂ are weighting coefficients (w₁ = 0.8, w₂ = 0.2) prioritizing measurement precision while maintaining real-time performance. The fitness function explicitly penalizes both measurement uncertainty and computational latency (Equation 11):

$$\begin{array}{c} F(\theta_{0},G)=\mathrm{\alpha U}(\theta_{0},G)+(1-\alpha )T(\theta_{0},G),\alpha =0.8 \end{array}$$ (11)

The algorithm converges within 50 iterations, yielding optimal parameters $\theta_{0}^{*}=44.3^{{}^{\circ}}$ and $G^{*}=4.7$ . Under this configuration, the measurement uncertainty of the analytic inverse mapping is reduced from 0.13 μm (manual tuning) to 0.08 μm (38% reduction), while keeping inference latency below 2 ms.

The methodology advances parameter optimization through a specifically adapted PSO, achieving automated, joint calibration of hardware parameters that are typically treated as independent tuning targets. A custom fitness function that simultaneously penalizes measurement uncertainty and computational latency is introduced, representing a novel approach in the context of this analytic inverse mapping problem. This joint optimization approach explicitly addresses and resolves the complex, non-linear interactions between $\theta_{0}$ and G , a limitation that traditional manual or sequential tuning methods fail to overcome. This balance between high precision and low latency is crucial (Ma et al., 2022).

### 6.4 Gaussian process regression for residual instrument error compensation

Even after PSO calibration, the analytic inverse mapping $x_{\text{analytic}}$ retains a systematic residual error that the Malus’s law model cannot account for. The primary sources of this residual are: (1) backlash and non-uniform pitch in the gear coupling, which introduce a nonlinear, direction-dependent deviation between the commanded and actual polarizer rotation angle; (2) spatial non-uniformity of the laser beam intensity profile, which causes the effective $I_{0}$ to vary slightly with polarizer orientation; and (3) non-ideal frequency response of the photoelectric ammeter at high sampling rates. These effects are instrument-specific and repeatable across measurement sessions, but they do not follow any known analytic form and therefore cannot be embedded as a physics constraint. A purely data-driven method is required, and the small size of the available calibration dataset—typically a few hundred reference measurements—further demands a model that generalises well from limited samples.

Gaussian Process Regression (GPR) is adopted as the residual corrector because it addresses both requirements simultaneously. A GPR model places a prior distribution over functions and updates it with observed data to yield a posterior that provides both a point estimate and a calibrated predictive variance for any new input. The GPR is trained on a dataset of residual pairs $\left\{(z_{i},r_{i})\right\}$ , where $z_{i}=(x_{\text{analytic},i},p_{i})$ is the two-dimensional input vector and $r_{i}=x_{\mathrm{ref},i}-x_{\text{analytic},i}$ is the measured residual error obtained from a reference standard during the offline calibration stage. The predictive mean at a new input $z^{*}$ gives the correction term $\delta x^{*}$ , and the predictive standard deviation $\sigma^{*}(z^{*})$ provides a direct uncertainty estimate for that correction, so that the final displacement and its associated uncertainty are given by (Equation 12):

$$\begin{array}{c} x_{\text{final}}=x_{\text{analytic}}+\delta x^{*},U_{\mathrm{GPR}}=k_{\mathrm{cov}}\cdot \sigma^{*}(z^{*}) \end{array}$$

(12)where $k_{\mathrm{cov}}$ = 2 corresponds to an approximately 95 % coverage interval. A Matérn-5 / 2 kernel (Chen et al., 2022) is selected for the covariance function because it assumes a degree of smoothness appropriate for instrument-induced mechanical errors, which are continuous but not infinitely differentiable. The kernel hyperparameters—length scale ℓ and signal variance $\sigma_{f}$ —are optimised by maximising the log marginal likelihood on the calibration dataset. Because the residual $\delta x^{*}$ is typically sub-micrometre in magnitude and varies smoothly with the operating point, the GPR model converges with fewer than 200 training points, resulting in a compact representation whose inference cost on the MCU is dominated by a single matrix–vector multiplication of size n (number of training points), well within the 2 ms latency budget. The integration of GPR uncertainty bounds into the measurement output also provides a natural mechanism for detecting anomalous operating conditions: when $\sigma^{*}(z^{*})$ exceeds a predefined threshold, the system flags the measurement as unreliable and initiates recalibration, as described in subsection 3.5.

### 6.5 Adaptive online recalibration strategy

In long-duration biomedical monitoring, slow environmental changes—including thermal drift of optical components, mechanical aging of the gear coupling, and gradual variation in laser output power—can shift the system’s operating point over time, causing both the PSO-calibrated parameters $\left(\theta_{0}^{*},G^{*}\right)$ and the offline-trained GPR model to become stale. To sustain accuracy under these conditions, an adaptive online recalibration strategy is incorporated into the measurement pipeline.

Recalibration is triggered by two complementary signals. The first is the sliding-window residual monitor, which tracks the RMS deviation between $x_{\text{final}}$ and the expected noise floor over 200 consecutive samples and triggers recalibration when this deviation exceeds three times the baseline standard deviation. The second is the GPR predictive uncertainty itself: when $\sigma^{*}(z^{*})$ exceeds a predefined threshold for more than 10 consecutive measurements, the system infers that the current operating point has drifted outside the domain covered by the training data and triggers recalibration proactively, before the residual error becomes large enough to affect the displacement output. The recalibration procedure operates at two levels simultaneously. At the hardware level, PSO performs a rapid local search of 10 iterations around the current $\left(\theta_{0}^{*},G^{*}\right)$ configuration and updates the parameters if fitness has degraded by more than 5%. At the model level, a batch of 50 fresh reference measurements is collected and appended to the GPR training set; the kernel hyperparameters are then re-optimised by maximising the updated log marginal likelihood. Because GPR hyperparameter optimisation involves only a low-dimensional gradient computation over the kernel parameters, this update completes in well under 100 ms and does not interrupt the measurement stream. This two-level mechanism ensures that both the analytic path and the GPR corrector remain jointly optimised throughout extended monitoring sessions.

The complete processing pipeline of the proposed system operates as follows. In the offline calibration stage, PSO determines the optimal hardware configuration $\left(\theta_{0}^{*},G^{*}\right)$ , after which the GPR model is trained on the resulting residual dataset and its kernel hyperparameters are optimised. In the online measurement stage, each intensity sample p is processed by the analytic inverse mapping to yield $x_{\text{analytic}}$ , which is then corrected by the GPR posterior mean to produce $x_{\text{final}}$ ; the GPR predictive variance simultaneously yields an uncertainty bound $U_{\mathrm{GPR}}$ for each measurement. The residual monitor and the GPR uncertainty threshold run continuously and trigger incremental recalibration whenever drift is detected. This architecture preserves the physical interpretability and computational efficiency of the analytic path, adds targeted data-driven correction only where the physical model is insufficient, and produces per-measurement uncertainty estimates fully consistent with the metrological reporting framework of this study.

## 7 Instrument innovation: deepening of interference and polarization principles and equipment improvement

In the preliminary stage of experimental teaching, systematic modifications were implemented to address the limitations of classical Michelson interferometers, integrating practical applications of micro-displacement measurement in clinical surgical microscopy and optical coherence tomography (OCT). By analyzing the mapping mechanism between the polarization angle-intensity response and micro-displacement, the physical basis for improving displacement measurement accuracy via polarization modulation technology was elucidated. Standardized procedures were established for photoelectric detector signal acquisition, micrometer handwheel operation, and data recording, with special emphasis on key technical requirements for environmental interference suppression and signal stability. Students were required to independently complete optical path calibration, integration and installation of polarization elements, and preliminary data collection, followed by establishing quantitative relationships among angle, displacement, and light intensity through image fitting.

## 8 Application innovation: high-precision measurement practices in medical settings

In response to the development orientation of New Medical Science, this study expands the application potential of high-precision micro-displacement measurement technology in the interdisciplinary field of medicine and engineering, with a particular focus on the practical integration of medical micro-displacement monitoring scenarios. The modified system is applied for the first time to the detection of clinical physiological parameters, providing a novel solution for real-time intraoperative monitoring and an original technical pathway for the development of new diagnostic and therapeutic equipment. Through the practice of the medicine-engineering integration project, students not only integrate interdisciplinary knowledge systems but also cultivate innovative thinking rooted in clinical needs, thereby supporting future technological in-novation and talent reserve in the field of medical devices.

## 9 Conclusion

This study successfully developed a modified Michelson interferometer system based on Malus’s law. By mechanically coupling the linear micro-displacement of the movable mirror to the angular variation of the polarizer and utilizing a photodetector to detect light intensity signals, the system achieves indirect and high-precision measurement of micro-displacement. This approach fundamentally overcomes the bottle-neck of traditional interferometric measurements limited by the coherent length of the light source, replacing inefficient manual fringe counting with automated photoelectric detection. To further unlock the precision potential of this physical foundation, three algorithmic innovations are introduced: PSO-based joint calibration that replaces manual tuning with automated optimization, analytic inverse mapping that preserves physical interpretability while enabling real-time computation, and GPR-based residual compensation that corrects instrument-specific errors and provides uncertainty bounds—a feature critical for medical applications where measurement reliability is paramount. An adaptive online recalibration mechanism further sustains long-term accuracy under real-world conditions.

Experimental results indicate that within the quasi-linear region of the Malus’s law curve, the measurement accuracy of the system is significantly superior to that of conventional methods, reaching up to 2.46 times higher. This fully validates its application potential in high-precision, wide-range micro-displacement measurement. This study not only provides a new technical pathway for micro-displacement monitoring in the biomedical field but also integrates the entire technological development process into teaching through the medicine-engineering integration project practice, offering a high-quality example for cultivating medical engineering talents with interdisciplinary innovation and practical capabilities.

In future work, efforts will be directed toward device miniaturization to facilitate portable point-of-care applications, along with algorithm lightweighting to enable embedded deployment. Further clinical validation will also be carried out in scenarios such as joint motion monitoring and respiratory waveform acquisition to evaluate the system’s long-term stability under real-world conditions.

## Data Availability

The original contributions presented in the study are included in the article/supplementary material, further inquiries can be directed to the corresponding author.
